# Evolutionarily conserved role of *hps1* in melanin production and blood coagulation in medaka fish

**DOI:** 10.1093/g3journal/jkac204

**Published:** 2022-08-09

**Authors:** Norimasa Iwanami, Yuka Ozaki, Hiyori Sakaguchi, Yuko Watanabe, Qi Meng, Kyoka Matsumoto, Tomohiro Suzuki, Kiyotaka Hitomi, Masaru Matsuda

**Affiliations:** Center for Bioscience Research and Education, Utsunomiya University, Utsunomiya 321-8505, Japan; Center for Bioscience Research and Education, Utsunomiya University, Utsunomiya 321-8505, Japan; Center for Bioscience Research and Education, Utsunomiya University, Utsunomiya 321-8505, Japan; Graduate School of Pharmaceutical Sciences, Nagoya University, Nagoya 464-8601, Japan; Graduate School of Pharmaceutical Sciences, Nagoya University, Nagoya 464-8601, Japan; Tochigi Girl's High School, Tochigi 328-0074, Japan; Center for Bioscience Research and Education, Utsunomiya University, Utsunomiya 321-8505, Japan; Graduate School of Pharmaceutical Sciences, Nagoya University, Nagoya 464-8601, Japan; Center for Bioscience Research and Education, Utsunomiya University, Utsunomiya 321-8505, Japan

**Keywords:** Hermansky–Pudlak syndrome, medaka fish, albinism, blood coagulation, melanin production, *hsp1*

## Abstract

Hermansky–Pudlak syndrome is an autosomal recessive disease characterized by albinism, visual impairment, and blood platelet dysfunction. One of the genes responsible for Hermansky–Pudlak syndrome, *hps1*, regulates organelle biogenesis and thus plays important roles in melanin production, blood clotting, and the other organelle-related functions in humans and mice. However, the function of *hps1* in other species remains poorly understood. In this study, we discovered albino medaka fish during the maintenance of a wild-derived population and identified *hps1* as the responsible gene using positional cloning. In addition to the specific absence of melanophore pigmentation, the *hps1* mutant showed reduced blood coagulation, suggesting that *hps1* is involved in clotting caused by both mammalian platelets and fish thrombocytes. Together, the findings of our study demonstrate that *hps1* has an evolutionarily conserved role in melanin production and blood coagulation. In addition, our study presents a useful vertebrate model for understanding the molecular mechanisms of Hermansky–Pudlak syndrome.

## Introduction

Hermansky–Pudlak syndrome (HPS) is an autosomal recessive disease that affects cytoplasmic organelles, such as melanosomes, platelet dense granules, and lysosomes, and results in albinism as well as breeding and lysosomal storage disorders (Hermansky and Pudlak 1959; Wei 2006). Among the 10 human HPS subtypes and the corresponding mouse subtypes with similar albinism phenotypes, it has been reported that *hps1* gene mutations are most often responsible for HPS in humans, with deficiency of the mouse ortholog causing a similar phenotype (Oh *et al.* 1996; Gardner *et al.* 1997; Wei 2006; Huizing *et al.* 2020).

HPS1 and HPS4 are components of the biogenesis of lysosome-related organelles complex (BLOC)-3 (Chiang *et al.* 2003; Martina *et al.* 2003; Nazarian *et al.* 2003), known as a guanine nucleotide exchange factor for RAB32 and RAB38 (Gerondopoulos *et al.* 2012). BLOC-3 and these RABs function in the forward traffic of cargo proteins to lysosome-related organelles (LROs; Wasmeier *et al.* 2006; Ambrosio *et al.* 2012; Bultema *et al.* 2012) and the retrograde traffic from LROs (Dennis *et al.* 2016). In melanotic cells, BLOC-3 is involved in the biogenesis of early melanosomes (Dell'Angelica *et al.* 2000).

In addition to mammals, some nonmammalian genetic models of HPS have been established (Bowman *et al.* 2019), including Xenopus models of HPS4 (Fukuzawa 2021) and HPS6 (Nakayama *et al.* 2017), and a zebrafish model of HPS5 (Daly *et al.* 2013). Although the genes were not identified, the zebrafish fade-out mutant has both ocular and blood clotting defects (Bahadori *et al.* 2006). In addition, the pinky zebrafish mutant has a loss of pigmentation and mutations in *hps1*, *ap3m2*, and *rabggta* (Hsu *et al.* 2013). The knockdown of *hps* genes in zebrafish has been reported to induce some HPS phenotypes, including renal protein loss (Schenk *et al.* 2019).

Medaka is a freshwater fish that has been widely used as a vertebrate model in genetic studies (Wittbrodt *et al.* 2002). Various mutant strains with disrupted pigmentation have been established (Kelsh *et al.* 2004), including strains with unpigmented melanophores in which *tyrosinase* and *pink-eyed dilution* (*oca2*) have been identified as the responsible genes (Koga *et al.* 1995; Fukamachi *et al.* 2004). In this study, we discovered an albino mutant medaka in our wild population that also displayed reduced thrombocyte clotting. Positional cloning identified a frameshift mutation in *hps1*, which suggests that the role of *hps1* in the biosynthesis of melanosome and platelet (thrombocyte) dense granules is conserved among vertebrates.

## Methods

### Fish maintenance

A wild Japanese medaka (*Oryzias latipes*) population originally collected in Tango, Kyoto prefecture was maintained as the Tango strain (strain ID: WS240) in the National Bioresource Project (NBRP) Medaka in Japan and categorized as a Southern strain. Albino fish identified in the Tango strain and their black siblings were maintained in temperature-controlled tanks (26°C) with a water circulating system under a 14 h light/10 h dark cycle. Albino medaka that emerged from the Tango strain were crossed with black medaka from the Tango strain. If offspring did not contain albino fish, the black Tango fish were considered to be wild type and the offspring were considered heterozygous. Albinism was judged by eye melanin defects 4 days postfertilization (dpf) under a stereo microscope with an upper light. Northern (*Oryzias sakaizumii*) inbred HNI-II strain (IB176) and Southern (*O. latipes*) strain OK-Cab (MT830) were also obtained from NBRP Medaka. This study was conducted in accordance with the ethical guidelines of the Utsunomiya University Animal Experimentation Committee and the experimental protocols were approved by the committee (Approval No. A20c-0012). This study is reported in accordance with ARRIVE guidelines (https://arriveguidelines.org).

### Positional cloning of the gene responsible for albinism

Albino/Tango medaka were crossed with HNI medaka for locus mapping. F1 fish were crossed with albino/Tango fish and genomic DNA was extracted from the whole body of each F2 embryo using a QuickGene DNA Tissue Kit (Kurabo, Osaka, Japan) from 6 dpf after black/albino determination. The DNA pools of 39 black and 35 albino embryos were used to determine the responsible linkage group using M-marker 2009 (Kimura and Naruse 2010). For fine mapping, we used self-made in/del markers for the DNA of 1,448 black and 1,332 albino embryos (2780 total). *hps1* was selected as a candidate gene from 14 genes located in the mapped locus. *Hps1* cDNA was sequenced to find the mutation, which was confirmed by PCR using genomic DNA. The primers used are listed in Supplementary Table 1.

### Genome editing

Two Alt-R CRISPR-Cas9 crRNAs targeting exon 4 of *hps1*, Alt-R CRISPR-Cas9 tracrRNA, and Cas9 protein were purchased from Integrated DNA Technologies (IDT; Singapore, Singapore). The sgRNA sequences are shown in Supplementary Table 1. RNA-protein complexes were prepared according to the manufacturer’s instructions. Glass capillaries with 1.0 mm outer diameter were pulled (temperature 62°C, force: 2 light weights and 2 heavy weights provided by the manufacturer) using a micropipette puller PC-10 (Narishige Instruments, Tokyo, Japan). The capillary was filled with 1–2 nL of each crRNA (final concentration 0.75 mM), tracrRNA (final concentration 1.5 mM), and 0.25 mg/ml Cas9 protein and then injected into 1-cell Cab embryos using a FemtoJet 4i (Eppendorf, Hamburg, Germany). G0 fish were crossed with wild-type Cab fish to establish the *hps1* mutant strains. *hps1^+/del13^* F1 fish were intercrossed and the embryos were observed at stage 31.

### RNA-seq

RNA was extracted from the whole bodies of 3 black (*hps1^+/+^*) and 3 albino adult medaka (*hps1^del14/del14^*) using Qiazol and an RNeasy Mini kit (Qiagen, Hilden, Germany). RNA-seq libraries were prepared using a Kapa Stranded mRNA-Seq Kit (Nippon Genetics, Tokyo, Japan). Six libraries (*n *=* *3) were mixed equally and sequenced by Macrogen Japan using a HiSeqX_Ten (151 bp, paired-end; Supplementary Table 2). Raw reads were trimmed using Trimmomatic (ver. 0.39; Bolger *et al.* 2014) and the remaining high-quality reads were mapped to the reference genome using HISAT2 (ver. 2.1.0; Pertea *et al.* 2016). The genomic DNA sequence of *O. latipes* from the National Center for Biotechnology Information (NCBI) database (ASM223467v1) was used as the reference genome. Genes were quantified using StringTie (ver. 1.3.4). Gene expression variation analysis was performed using Ballgown (ver. 2.18.0).

### Thrombocyte observation

Blood cells were harvested from 3-month-old medaka, smeared on glass slides, and allowed to dry. May-Giemsa staining was carried out with dye solution (Wako, Osaka, Japan) as per the manufacturer's instructions.

### Blood clot formation

Blood clot formation was analyzed in 6-month-old medaka as described previously (Watanabe *et al.* 2021). Briefly, blood was collected in the presence of ethylenediaminetetraacetic acid (EDTA) to capture calcium ions and then incubated with an excess of calcium.

### Immunoblotting

Medaka blood samples were analyzed by immunoblotting as described previously (Watanabe *et al.* 2021). Blood lysate samples from each fish were subjected to SDS polyacrylamide gel electrophoresis (SDS-PAGE) for Coomassie Brilliant Blue (CBB) staining and immunoblotting analyses. For immunoblotting, proteins were transferred to a membrane and incubated with antifibrinogen gamma primary antibody (prepared recombinant protein for fibrinogen gamma subunit as the antigen, Meng *et al.* unpublished data) and antirabbit IgG conjugated with peroxidase (Jackson ImmunoResearch Laboratories, West Grove, PA, USA) as the secondary antibody. Signals were developed using a chemiluminescent reagent (Thermo Fisher Scientific, San Jose, CA, USA).

## Results

### Establishment of a strain with defective melanophore pigmentation from a wild medaka population

**Fig. 1.**
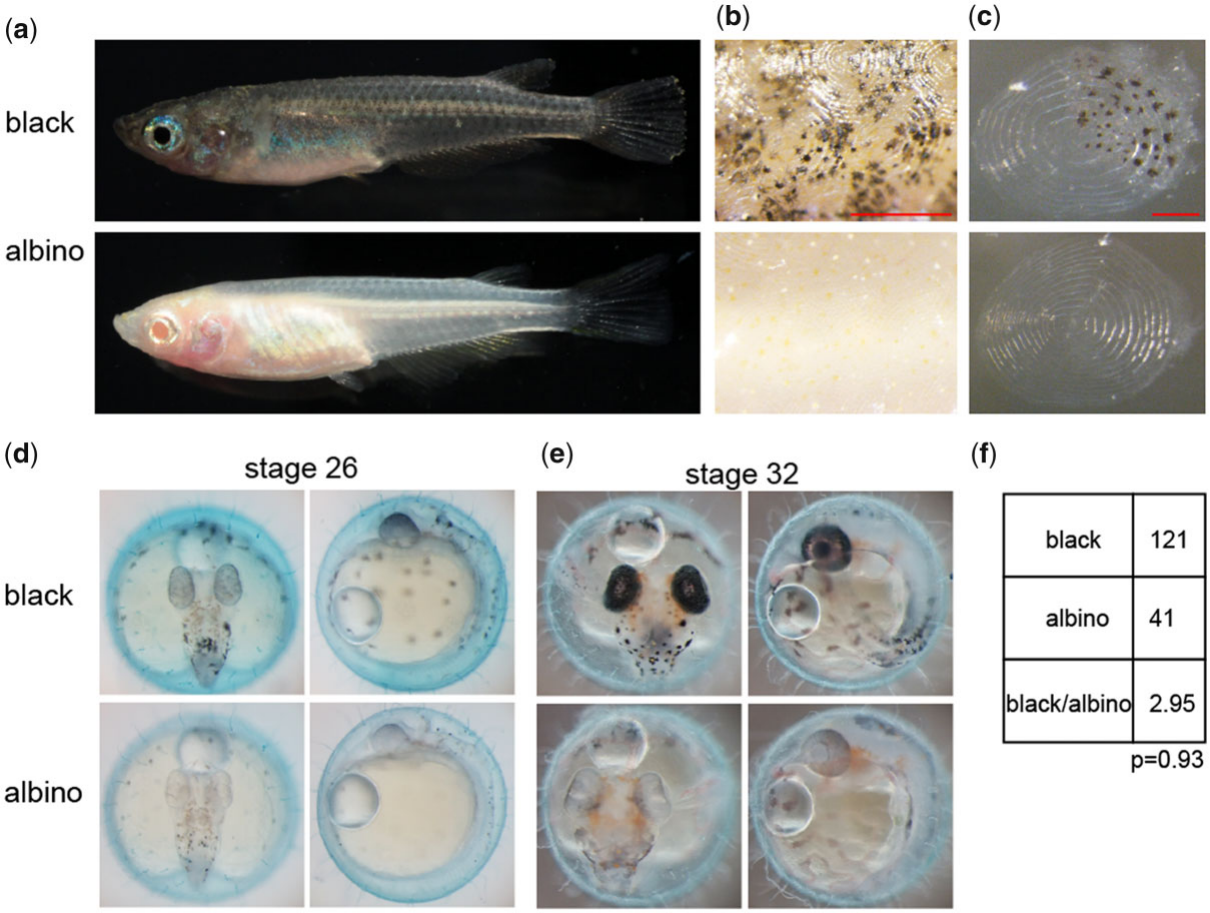
Melanophore pigmentation is defective in wild-derived albino medaka. a) Appearance of black (top) and albino (bottom) medaka siblings 4 months posthatching. b) Magnified view of the trunk of black (top) and albino (bottom) fish. Scale bar; 500 µm. c) Trunk scale from black (top) and albino (bottom) fish. Scale bar; 200 µm. d, e) Embryo appearance of stage 26 (d) and stage 32 (e) black (top) and albino (bottom) fish. Dorsal (left) and lateral (right) views. f) Frequency of albino embryos from heterozygote intercross. Chi-squared test.

The NBRP Medaka keeps wild populations of *O. latipes* collected in the late 1980s from 90 locations in Japan, China, Taiwan, and Korea (Takehana *et al.* 2003, 2004). During the repetitive intercrossing of the population derived from Tango, Kyoto prefecture (strain ID: WS240), albino fish emerged. Melanophores were specifically affected in adult albino fish, which lacked skin and retinal pigment epithelium pigmentation, but retained iridophores in the iris, gill lid, and peritoneum (Fig. 1a). Enlarged trunk images revealed the absence of melanophores, but not of xanthophores and leucophores, in the skin of albino fish (Fig. 1b), whereas their scales lacked both melanophores and xanthophores (Fig. 1c). Defective melanin pigmentation was observed in albino embryos at stage 26 (Iwamatsu 2004), when pigmentation started in the eyes and dorsal skin of wild-type embryos (Fig. 1d). By stage 32, melanophore pigmentation was affected in the eyes and skin of albino embryos, but not iridophore pigmentation (Fig. 1e). Together, these findings suggest that our albino medaka specifically lacks melanophore pigmentation, consistent with the phenotypes of previously reported *tyrosinase* and *oca2* mutant medaka (Koga *et al.* 1995; Fukamachi *et al.* 2004; Kelsh *et al.* 2004). Intercrossing black heterozygous fish generated albino offspring at a rate of approximately 25% (Fig. 1f), suggesting that the albino phenotype was recessive and was caused by a single genome locus.

### Identification of *hps1* as a gene responsible for albinism

**Fig. 2.**
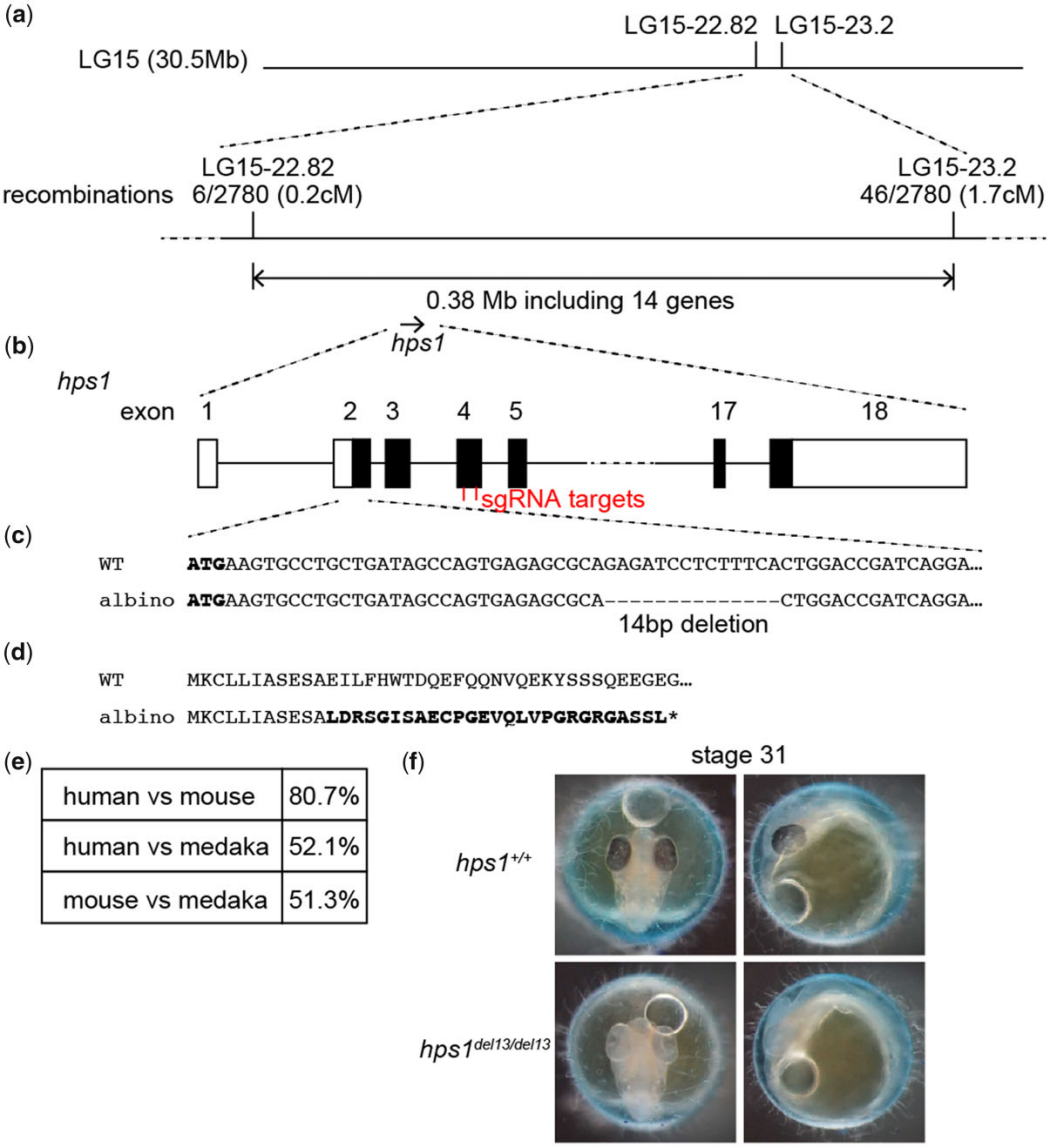
Identification of the gene responsible for albinism. a) Mapping of the locus responsible for the albino phenotype. Recombination rates and genetic distances between the markers and the responsible locus are shown. b–d) *hps1* gene structure and the mutation in albino medaka. b) Exon/intron structure of the *hps1* gene. Coding exons are indicated as closed boxes. Untranslated exons are indicated as open boxes. sgRNA targets in exon 4 for genome editing are indicated. c) Fourteen base pair deletion in exon 2 in albino medaka. d) Deduced amino acid sequences of wild-type and albino Hps1 proteins. Bold letters indicate unrelated amino acids. * indicates the stop codon. e) The percentages of identical amino acids between the human, mouse, and medaka hps1 proteins. f) Albino phenotype caused by *hps1* genome editing. Dorsal (left) and lateral (right) views of stage 31 *hps1^+/+^* and *hps1^del13/del13^* embryos.

We investigated the genetic basis of the albino phenotype by performing linkage analysis using M-marker 2009 (Kimura and Naruse 2010) with the DNA pools of 39 black and 35 albino embryos derived from (albino/Tango × HNI-II) × albino/Tango crosses to map the responsible locus on LG15 (data not shown). Fine mapping using self-made markers for LG15 placed the responsible locus between 2 markers: LG15-22.82 (0.2 cM from responsible locus) and LG15-23.2 (1.7 cM from responsible locus; Fig. 2a). This genomic region between these markers (0.38 Mb) contained 14 genes (Supplementary Table 3) including *hps1*, which is a paralog of human and mouse genes whose mutations have been reported to be responsible for albinism (Oh *et al.* 1996; Feng *et al.* 1997). Therefore, we searched for mutations in the *hps1* gene in the albino fish (Fig. 2b). RNA-seq analysis of the whole bodies of adult black and albino medaka (Supplementary Tables 4 and 5) identified a 14-bp deletion in exon 2 of the *hps1* transcript (Fig. 2c), which presumably causes a frameshift after the 11th amino acid (Fig. 2d). This deletion was confirmed in albino medaka using genomic PCR and the amino acid sequence of medaka Hps1 was found to be 52% and 51% identical to human and mouse orthologs, respectively (Fig. 2e).

In order to functionally confirm that *hps1* was the gene responsible for albinism in medaka, genome editing was carried out using the CRISPR/Cas9 system targeting exon 4 of *hps1*. A mutant line with a 13-bp deletion in *hps1* was established (Supplementary Fig. 1) and the homozygous form of this frameshift mutant was found to mimic the albino phenotype (Fig. 2f), confirming that *hps1* mutation was responsible for the observed albino medaka.

### Low competitiveness in body growth of albino medaka

**Fig. 3.**
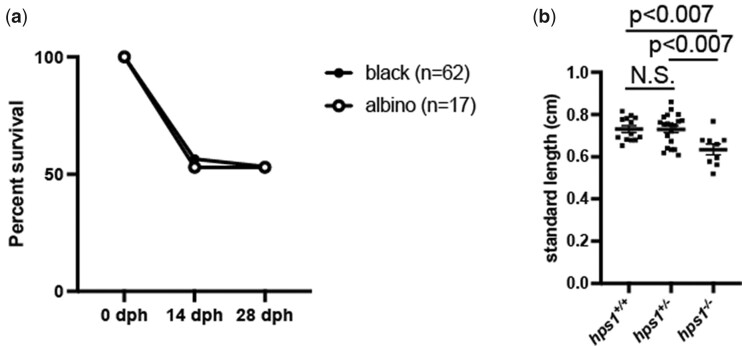
Low competitiveness in body growth of albino medaka. a) The percentages of surviving black (closed circles) and albino (open circles) fish at the indicated days posthatching (dph) (left *y*-axis). b) Standard lengths of each *hps1* genotype at 28 dph. Data represent the mean ± SEM. Unpaired 2-tailed *t*-test.

Next, we investigated whether our albino medaka had survival disadvantages compared to their black siblings. When hatched albino and black siblings derived from *hps1^+/−^* intercrosses were reared in the same tank, their survival rates were comparable (Fig. 3a); however, *hps1^−/−^* albino fish were significantly shorter in length than *hps1^+/+^* and *hps1^+/−^* black siblings (Fig. 3b). This result suggests that albino medaka display low competitiveness in body growth even in the laboratory environment. RNA-seq analysis of the whole bodies of adult black and albino medaka revealed that *crystallin* genes were differentially expressed in albino fish (Supplementary Fig. 2), suggesting that their reduced growth may be related to their eyesight.

### Albino medaka display less efficient thrombocyte aggregation

**Fig. 4.**
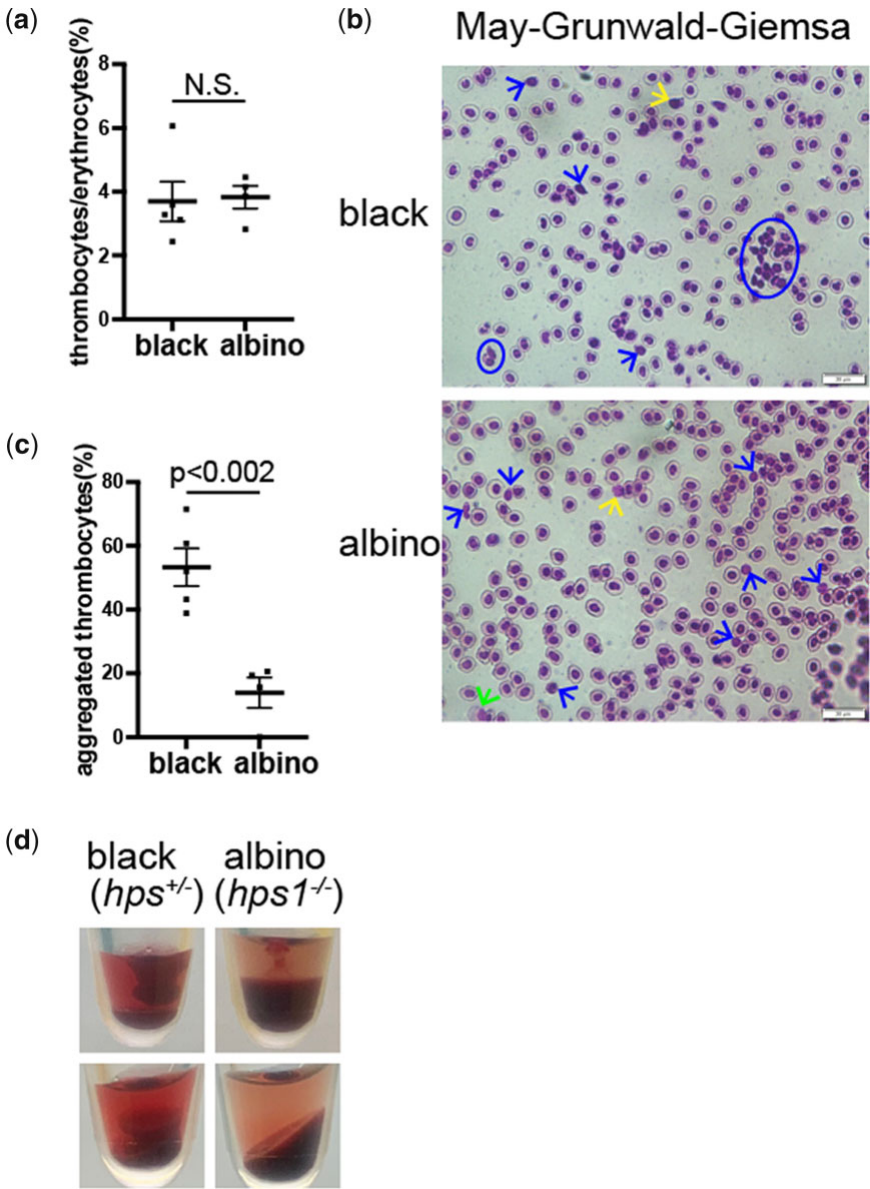
Reduced aggregation activity of albino thrombocytes. a) The percentages of thrombocytes vs erythrocytes in peripheral blood smears from black and albino medaka. Each dot represents one individual. Data represent the mean ± SEM. Unpaired 2-tailed *t*-test. b) The representative images of blood smears from black and albino fish stained with May–Grunwald–Giemsa. Blue circle: aggregated thrombocytes. Blue arrow: thrombocyte. Yellow arrow: lymphocyte. Green arrow: large mononuclear cell. c) The percentages of aggregated thrombocytes among all thrombocytes. Each dot represents one individual. a, c) Data represent the mean ± SEM. Unpaired 2-tailed *t*-test. d) Blood coagulation pattern in adult medaka. Images were taken before (top) and after (bottom) centrifugation.

Patients with HPS and *Hps1*-deficient *pale ear* (*ep*) mutant mice exhibit a decrease in the number of platelet dense granules, leading to prolonged bleeding time (Novak *et al.* 1984; Meng *et al.* 2015; Sharda *et al.* 2015). Therefore, we investigated the aggregation efficiency of thrombocytes in peripheral blood from our albino and black medaka using May-Giemsa staining. Although the ratio between the numbers of thrombocytes and erythrocytes was comparable (Fig. 4a), albino fish had a significantly lower percentage of aggregated thrombocytes than their black siblings (Fig. 4, b and c). In addition, blood clot formation was reduced in albino peripheral blood (Fig. 4d), indicating that less efficient blood coagulation is a conserved feature of *hps1*-deficient albino vertebrates.

To clarify the mechanism underlying the blood coagulation defect in albino medaka, we analyzed fibrinogen levels as a major coagulant factor in peripheral blood using immunoblot analysis. The levels of fibrinogen gamma protein were approximately 30% lower in homozygous *hps1* mutants than in heterozygotes (Supplementary Fig. 3), which may affect the blood coagulation efficiency of fibrin. Notably, RNA-seq analysis of the whole bodies of black and albino adult medaka revealed that the expression of fibrinogen family members was decreased in albino fish (Supplementary Fig. 4).

## Discussion

In this study, we established a novel albino medaka strain derived from a wild population and identified the responsible gene as *hps1*. Medaka have been used to study pigmentation for over 100 years (Ishiwara 1917; Aida 1921) and thus many pigmentation mutants have been established (Kelsh *et al.* 2004). For instance, the *tyrosinase* mutant displays melanophore defects, similar to our albino phenotype (Koga *et al.* 1995), while the *oca2* mutant has milder melanophore formation defects (Fukamachi *et al.* 2004). Although many studies have investigated melanophores in medaka (Hashimoto *et al.* 2021), this is the first to report that *hps1* mutation causes an albino phenotype in medaka. The findings also demonstrate that the survival rate of albino fish was comparable to that of their black siblings; however, the shorter body length of albino fish suggests low competitiveness in getting food even in the laboratory environment (Fig. 3). The reduced competitiveness in body growth could be related to poor eyesight in *hps1* mutant medaka. Although we did not specifically examine the eyesight of the albino medaka in this study, the homozygotic mutants generated using our panels of wild strains may not survive in nature. From these findings, it can be inferred that the low competitiveness in body growth could have led to poor survival of *hps1* mutant medaka in the wild.

We also demonstrated that the function of *hps1* is evolutionally conserved in vertebrates and accordingly established a fish model of HPS. Although previous studies have reported that *hps1* knockdown in zebrafish causes a severe renal phenotype with pericardial effusion, yolk sac edema, and protein loss (Schenk *et al.* 2019), and have described pinky zebrafish mutant lacking pigmentation with mutations in some genes including *hps1* (Hsu *et al.* 2013), our study is the first to report a genetic model fish without *hps1* function. In addition, we showed that *hps1* specifically affects the production of melanophores rather than other pigments (Fig. 1), consistent with previously reported albino mutant lines such as the *tyrosinase* mutant (Kelsh *et al.* 2004). The phenotypic similarity of the *tyrosinase* mutant and *hps1* mutant is probably due to the functional relevance of these 2 proteins as shown in mouse melanocytes; BLOC-3 including Hps1 is a guanine nucleotide exchange factor of Rab32/Rab38 (Gerondopoulos *et al.* 2012), which mediate transport of tyrosinase from a perinuclear compartment to the melanosome (Wasmeier *et al.* 2006).

Furthermore, we found that the *hps1* mutant albino medaka exhibited blood coagulation defects (Fig. 4), as seen in patients with HPS and corresponding mouse models. Although we found that fibrinogen expression was reduced in *hps1* mutant fish (Supplementary Figs. 3 and 4), further studies are required to determine the molecular mechanisms underlying blood coagulation deficiencies in our *hps1* mutant medaka.

In summary, we believe that the *hps1* mutant medaka model generated in this study could be useful for future drug screening and bioimaging studies to identify the molecular mechanisms underlying HPS.

## Supplementary Material

jkac204_Supplementary_Fig_S1

jkac204_Supplementary_Fig_S2

jkac204_Supplementary_Fig_S3

jkac204_Supplementary_Fig_S4

jkac204_Supplementary_Table_S1

jkac204_Supplementary_Table_S2

jkac204_Supplementary_Table_S3

jkac204_Supplementary_Table_S4

jkac204_Supplementary_Table_S5

## Data Availability

The datasets generated during the current study are available in the DNA Data Bank of Japan (DDBJ) database (accession number DRA013704) repository (https://ddbj.nig.ac.jp/search?query=%22DRA013704%22; accessed 2022 Aug 13). Supplemental material is available at G3 online.
