# Supplementary figures and images for: Evolutionarily conserved role of hps1 in melanin production and blood coagulation in medaka fish
Source: G3 (Bethesda). 2022 Aug 9;12(10):jkac204. doi: 10.1093/g3journal/jkac204 (PMC9526055; doi:10.1093/g3journal/jkac204)

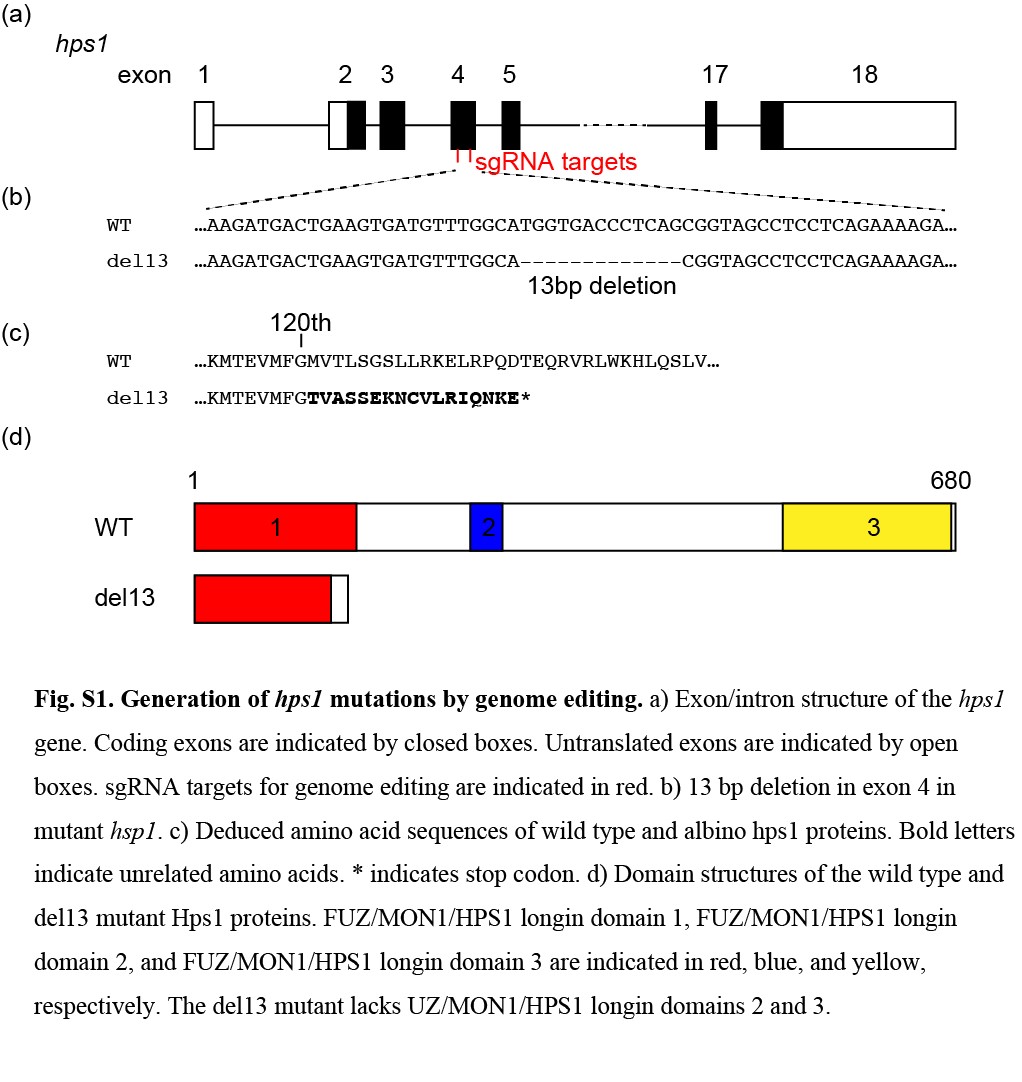

Supplement: jkac204_Supplementary_Fig_S1 [file jkac204_supplementary_fig_s1.jpeg]

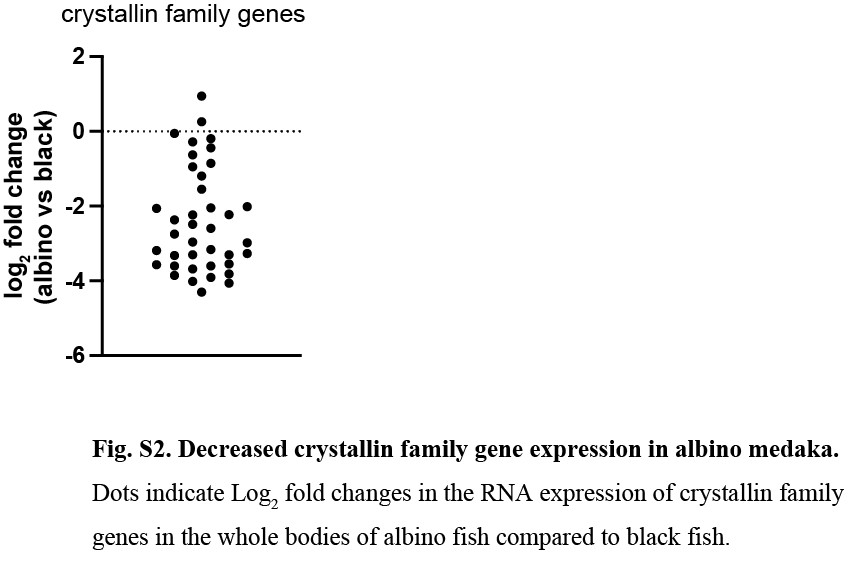

Supplement: jkac204_Supplementary_Fig_S2 [file jkac204_supplementary_fig_s2.jpeg]

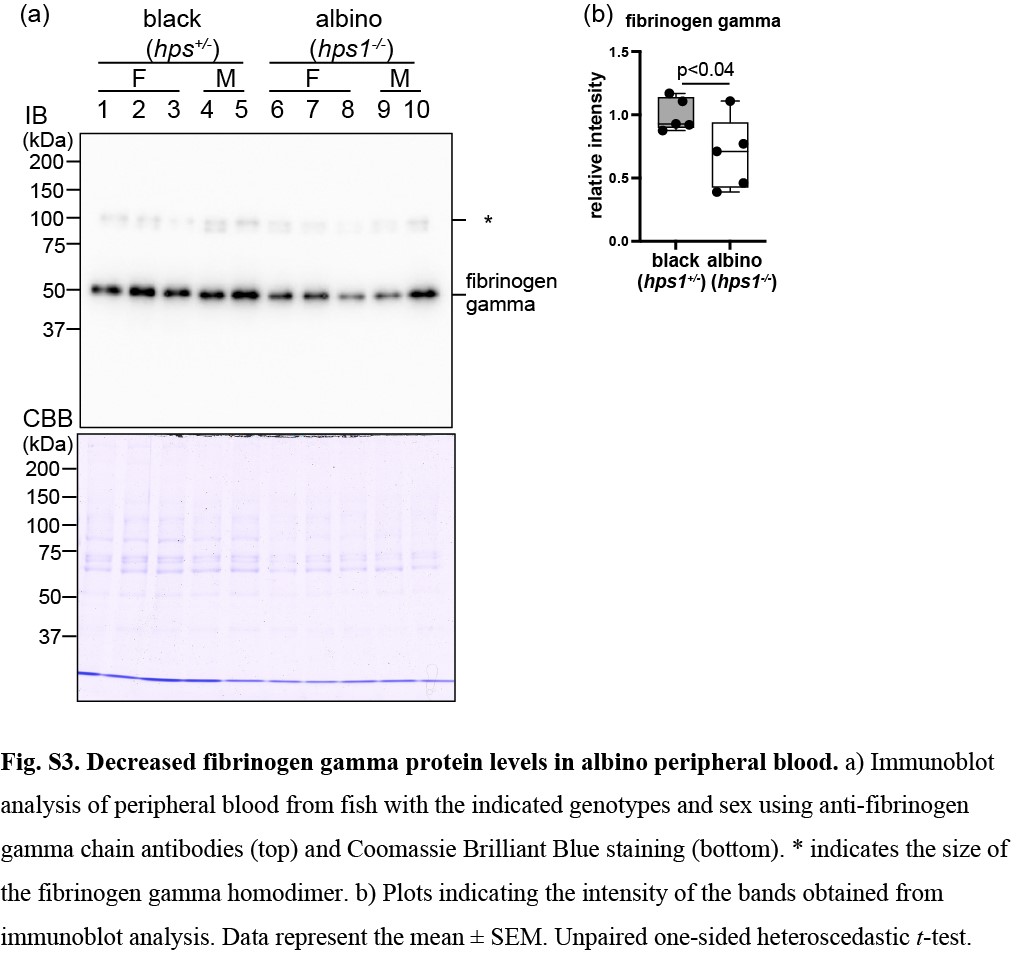

Supplement: jkac204_Supplementary_Fig_S3 [file jkac204_supplementary_fig_s3.jpeg]

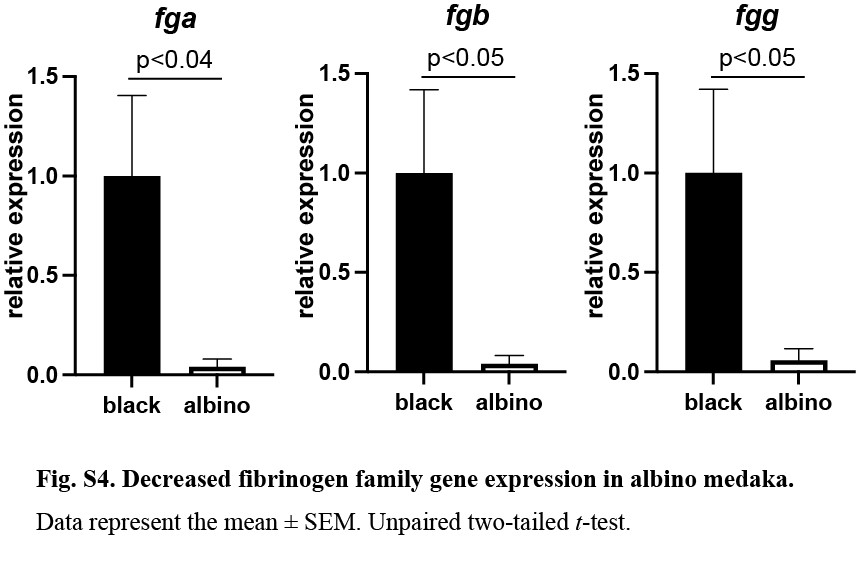

Supplement: jkac204_Supplementary_Fig_S4 [file jkac204_supplementary_fig_s4.jpeg]
