# Supplementary material for: Evolutionarily conserved role of hps1 in melanin production and blood coagulation in medaka fish
Source: G3 (Bethesda). 2022 Aug 9;12(10):jkac204. doi: 10.1093/g3journal/jkac204 (PMC9526055; doi:10.1093/g3journal/jkac204)
Supplement: jkac204_Supplementary_Table_S2 [file jkac204_supplementary_table_s2.docx]

Table S2 Number of sequencing reads.

|  | **Number of raw reads** | **Number of high-quality reads** |
| --- | --- | --- |
| **Black** |  |  |
| **Black1-read1** | 17,976,426 | 11,535,870 |
| **Black1-read2** | 17,976,426 | 11,535,870 |
| **Black2-read1** | 18,292,824 | 11,935,443 |
| **Black2-read2** | 18,292,824 | 11,935,443 |
| **Black3-read1** | 20,487,678 | 12,935,471 |
| **Black3-read2** | 20,487,678 | 12,935,471 |
| **Albino** |  |  |
| **Albino1-read1** | 18,491,815 | 12,680,658 |
| **Albino1-read2** | 18,491,815 | 12,680,658 |
| **Albino2-read1** | 18,569,374 | 12,655,649 |
| **Albino2-read2** | 18,569,374 | 12,655,649 |
| **Albino3-read1** | 16,712,459 | 10,919,441 |
| **Albino3-read2** | 16,712,459 | 10,919,441 |
